# Novel configurations of type I-E CRISPR-Cas system in *Corynebacterium striatum* clinical isolates

**DOI:** 10.1007/s42770-022-00881-4

**Published:** 2022-12-07

**Authors:** Juliana Nunes Ramos, Paulo Victor Pereira Baio, João Flávio Carneiro Veras, Érica Miranda Damásio Vieira, Ana Luiza Mattos-Guaraldi, Verônica Viana Vieira

**Affiliations:** 1 Laboratório de Difteria E Corinebactérias de Importância Clínica (LDCIC), Faculdade de Ciências Médicas, Universidade Do Estado Do Rio de Janeiro, Av. 28 de Setembro, 87, Fundos, 3º Andar, Vila Isabel, Rio de Janeiro, RJ Brazil; 2 Laboratório Químico-Farmacêutico Do Exército Brasileiro (LQFEx), Ministério da Defesa, Brasília, Brazil; 3 Laboratório Interdisciplinar de Pesquisas Médicas (LIPMED), Instituto Oswaldo Cruz, Fundação Oswaldo Cruz, Rio de Janeiro, Brazil

**Keywords:** CRISPR-Cas, *Corynebacterium striatum*, Multidrug-resistant, Health care infections, Emergent pathogen

## Abstract

Clustered regularly interspaced short palindromic repeats (CRISPR) are a prokaryotic adaptive immune system that, through Cas proteins, promote the degradation of foreign nucleic acids such as phages and plasmids. We analyzed 10 genomes of *Corynebacterium striatum* clinical isolates from a public hospital in Rio de Janeiro, Brazil, the most emergent multidrug-resistant *Corynebacterium* species. All isolates were submitted to antimicrobial susceptibility testing. The occurrence and diversity of the CRISPR system were investigated by bioinformatics tools. Our analysis revealed that the isolates exhibited type I-E gene arrangements, and 3 more multidrug-resistant isolates, alternative type I-E gene arrangements, showing a divergent gene arrangement within the *cas* operon. Phylogenetic analysis of the *cas1* gene of this type I-E CRISPR-Cas system alternative arrangement, termed here type I-E’, showed a cluster in a distinct clade of the type I-E CRISPR-Cas system. The systems’ guanine-cytosine (GC) content is lower than the genomic DNA’s GC content, and mobile genetic elements were found in some isolates near the CRISPR-Cas system. Most CRISPR spacers are unknown indicating that there is a reservoir of unexplored corynebacteriophages and plasmids. Some spacers showed perfect homologies with phage and plasmid sequences. Intact phage regions were found in 3 of our isolates, ranging from 9.1 to 43.8 kb, with regions showing similarity to *Rhodococcus* and *Corynebacterium* phages. Our results may contribute to research about the CRISPR-Cas system diversity in *C. striatum*, where there are no published data to date.

**Supplementary Information:**

The online version contains supplementary material available at 10.1007/s42770-022-00881-4.

## Introduction

*Corynebacterium* species are widely distributed in the environment and are part of the skin and mucosal microbiota [1, 2]. *Corynebacterium striatum* is a Gram-positive rod, non-sporulating recognized as a true pathogen in specific circumstances when isolated from patients with chronic diseases, indwelling medical devices, and several samples from sterile body sites [3–5]. *C. striatum* has been cited in several reports as a multidrug-resistant (MDR) health care infection pathogen, including septicemia, valvular damage, pulmonary infection, meningitis, endocarditis, osteomyelitis, and other invasive infections [1, 3, 6, 7]. Clonal MDR *C*. *striatum* has been affecting both immunocompromised and immunocompetent patients [1, 3, 6]. Multidrug resistance and patient-to-patient transmission have also been reported [3, 6].

The prokaryotic CRISPR (Clustered Regularly Interspaced Short Palindromic Repeats) system resembles an immunity system to host cells against invading nucleic acids and they represent a barrier to recombination. This system has been detected in bacteria and archaea. CRISPR-associated proteins (Cas) consist of a combination of Cas effector proteins that are involved in target DNA or RNA cleavage, CRISPR loci transcript processing, and novel spacer integration [8–11]. The CRISPR system uses mechanisms divided into three stages, the first being the adaptation phase, when a novel spacer is acquired from an invading nucleic acid. At this stage, there are Cas1 and Cas2 that form a complex involved in the adaptation step and are conserved in almost all CRISPR-Cas types. The second stage is the expression phase, where the CRISPR locus is transcribed into pre-crRNA, and *cas* genes are expressed and involved in pre-crRNA processing and in mature short crRNAs that form a complex with these genes. The last step is the interference phase, where invading nucleic acids will be recognized and cleaved by crRNA-Cas complexes [12, 13].

The CRISPR-Cas system prevents the spread of plasmids and bacteriophages and limits horizontal gene transfer by these mobile genetic elements [14]. In many bacterial species, antibiotic resistance is mediated by the acquisition of genes from plasmids and transposons. The presence of the CRISPR-Cas system and the acquisition of antibiotic resistance genes may have an inverse correlation. Studies have shown a significant association between antibiotic resistance in *Enterococcus faecalis* and the absence of the CRISPR-Cas system [15]. However, in a study performed on *Escherichia coli*, CRISPR-Cas system appears to ineffectively block plasmid dissemination and antibiotic resistance [16].

CRISPR and associated *cas* genes have been detected in some *Corynebacterium* species, such as *Corynebacterium diphtheriae* [8], *Corynebacterium bovis* [17], *Corynebacterium pseudotuberculosis* [18], *Corynebacterium ulcerans* [19], and *Corynebacterium urealyticum* [20]. In other *Corynebacterium* species, CRISPR-Cas system studies are scarce, except for *Corynebacterium glutamicum*, which is an important metabolite producer in the biotechnology industry [21]. The type I-E CRISPR-Cas system was found in most corynebacteria. In the pathogen that causes diphtheria diseases, *Corynebacterium diphtheriae*, type II-C system and type I-E variant system were found [8]. To date, there are no published data on diversity about the *C. striatum* CRISPR-Cas system, an opportunistic hospital-associated pathogen. In this study, we explored 10 *C. striatum* genomes isolated from a nosocomial outbreak in the city of Rio de Janeiro, Brazil, and 21 *C. striatum* genomes available at NCBI were analyzed for the presence and characterization of the CRISPR-Cas system and their spacers.

## Methods

### Bacterial isolates

We analyzed 10 *C. striatum* genomes, deposited at NCBI (Table 1), isolated from nosocomial outbreaks that occurred at a public university hospital in the city of Rio de Janeiro, Brazil, for 42 months (January 2009 to February 2013). In addition to the 10 genomes, we have included 21 *C. striatum* genomes, available at GenBank/NCBI, for comparative purposes (Supplementary Table 1).

**Table 1:** Details of CRISPR-Cas loci in Brazilian *C. striatum* isolates

Isolates^a^	Isolation date^a^	Isolation sites^a^	PFGE profiles^a^	CRISPR-Cas system (number of spacers)	GenBank access number
1954	October 2009	Surgical wound secretion	IV	–	PGGF01000000.1
1961	December 2009	Urine	III	I-E (15)	LAYR01000000.1
2023	August 2009	Blood	I	I-E (18) I-E’ (9)	LBCN01000000.1
2038	September 2009	Blood	II	I-E (51) I-E’ (59)	PGGG00000000.1
2130	August 2010	Blood	V	I-E (20)	NRIL00000000.1
2230	February 2011	Blood	VI	I-E (44) I-E (47)	LTBF00000000.1
2237	March 2011	Blood	VI	I-E (47)	NRIM01000000.1
2296	July 2011	Central venous catheter	VII	I-E (100)	NRIN00000000.1
2308	August 2011	Blood	I	I-E (117) I-E’ (58)	NRIO00000000.1
2425	August 2012	Blood	IX	I-E (55)	NRIP00000000.1

^a^According to Ramos et al. [4]

### Antimicrobial susceptibility testing

Antimicrobial susceptibility profiles were determined by minimum inhibitory concentration (MIC) using Etest strips on cation-adjusted Mueller–Hinton agar supplemented with 5% sheep blood using inoculum equivalent to a 0.5 McFarland standard. Interpretation of penicillin, ciprofloxacin, moxifloxacin, vancomycin, clindamycin, tetracycline, linezolid, and rifampin values was performed according to the current BrCAST/EuCAST guideline [22] (Table 2). MDR was defined as acquired non-susceptibility to at least one agent in three or more antimicrobial categories [23].

### Characterization of CRISPR-Cas diversity

CRISPRCasFinder^1^ (version CRISPR-Cas +  + 1.1.2) was used to identify the CRISPR-Cas system of genomes. CRISPR arrays with low evidence, equal to 0 or 1, were not included in the analyses [24]. CRISPR-Cas cassette type was determined following the nomenclature and classification described by Makarova et al. [11]. CRISPR array spacers were extracted from CRISPRFinder outputs. Spacer sequences were analyzed for their identity: at the CRISPRTarget^2^ database, which contained A CLAssification of Mobile Genetic Elements (ACLAME), Genbank-Phage, RefSeq-Plasmid, RefSeq-Viral, IslandViewer, PHAST and Community Cyber infrastructure for Advanced Microbial Ecology Research & Analysis (CAMERA) sequences, and the cut-off score was the default parameter value [25]; and at the ViroBLAST^3^ server, version 2.6 against viral databases using default parameters [26] and against spacers databases in the CRISPR-Cas +  + database^1^ with E-value = 0.01 [24]. Spacer hits were selected from the ViroBlast, CRISPRTarget, and CRISPR-Cas +  + databases with a cut-off identity cover according to Sangal et al. [8]. Direct repeat conservation was represented by WebLogo,^4^ version 2.82 [27]. Prophage sequences were identified from assembled contigs using the PHASTER^5^ webserver (PHAge Search Tool Enhanced Release) [28].

### Core genome and phylogenetic analysis

Prokka,^6^ version 1.14.6, was used for whole-genome annotation to produce standards-compliant GFF3 output files required for pangenome calculation [29]. The annotated gene repertoires of the studied genomes were grouped using Roary,^7^ version 3.13.3 using the parameters as follows: –mafft; -i 60 to calculate pan-genome and core-genome [30]. A neighbor-joining (NJ) phylogenetic tree was constructed from *C. striatum* core genome sequence alignment using Mega X^8^ [31]. NJ trees were also generated from nucleotide sequence alignments of *cas* genes and direct repeats consensus of CRISPR-Cas systems of all isolates by p-distance with 500 iterations for bootstrap. Geneious,^9^ version 2021.2.2, was used to assess Cas protein conservation by multiple sequence global alignment (Needleman-Wunsch) with standard parameters.

## Results

### Antimicrobial resistance profiles

Table 2 shows antimicrobial susceptibility profiles of 10 *C. striatum* isolates. Eight *C. striatum* isolates showed non-susceptibility to at least one agent in three or more antimicrobial categories and were identified as MDR pathogens. Isolates no. 1954 and 1961 were susceptible to all antimicrobials tested and showed intermediate susceptibility to ciprofloxacin, according to the BrCAST/EuCAST guideline [22]. Isolates no. 2023, 2038, and 2308 have an additional CRISPR-Cas system, termed here type I-E’ CRISPR-Cas, and were susceptible only to vancomycin, tetracycline, and linezolid. Our isolates showed resistance to penicillin (70%), ciprofloxacin (100%), moxifloxacin (80%), clindamycin (80%), and rifampin (30%). All isolates were susceptible to vancomycin, linezolid, and tetracycline.

**Table 2:**
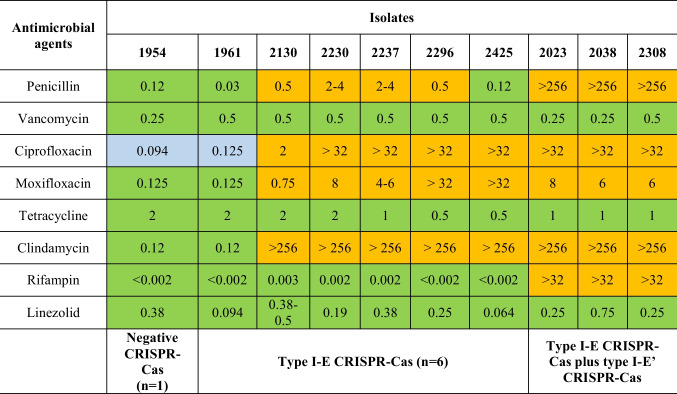
Minimum inhibitory concentration and antimicrobial susceptibility profiles of 10 *Corynebacterium striatum* isolates from blood (*n* = 7), central venous catheter (*n* = 1), urine (*n* = 1), and surgical wound secretion (*n* = 1) infections

According to the BrCAST guideline (2021), orange is resistant, blue is intermediate, and green is susceptible to antimicrobial agents

Isolation sites: surgical wound secretion (1954); urine (1961); blood (2023, 2038, 2130, 2230, 2237, 2308, and 2425); and central venous catheter (2296)

### CRISPR-Cas system diversity and arrangement in *C. striatum*

**Fig. 1:**
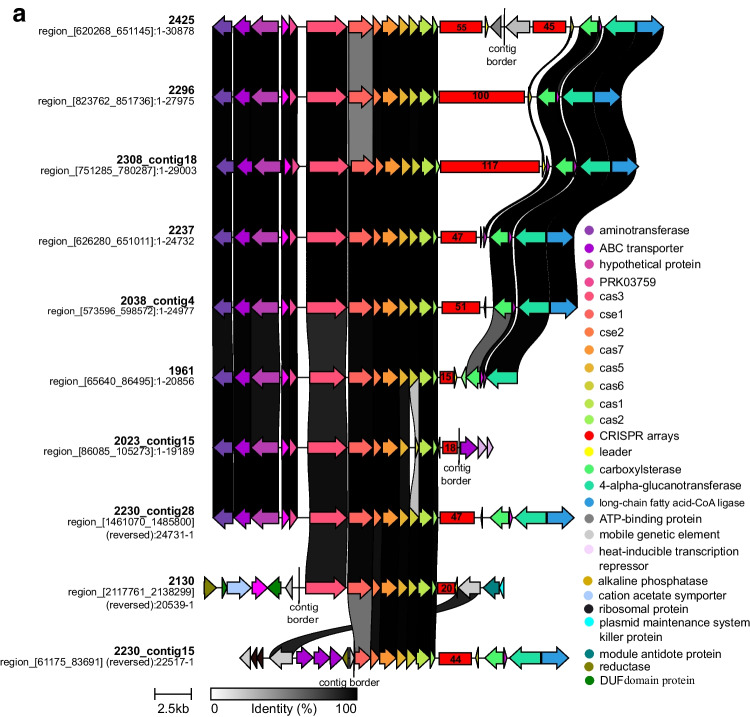

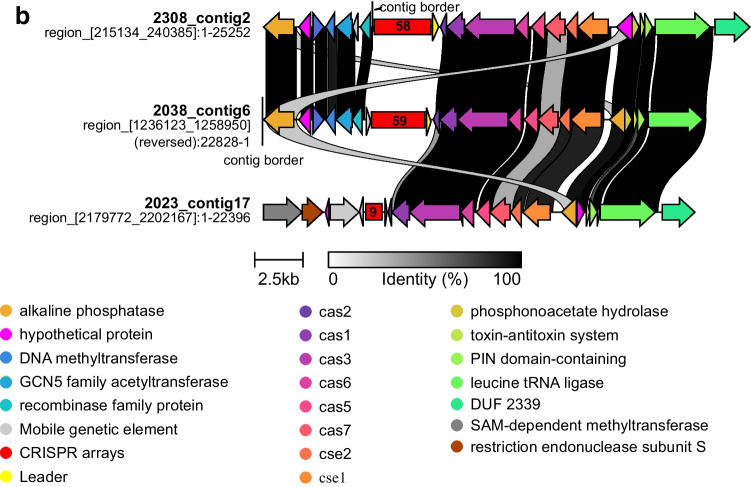
Type I-E CRISPR-Cas system (**a**) classic configuration and alternative gene arrangement named type I-E’ (**b**) found in Brazilian *C. striatum* isolates. The similarity scale between the *C. striatum* isolate CRISPR-Cas systems is represented below, ranging from 0 to 100% (white to black). The numbers in the red box indicate the quantity of spacer sequences. Isolates bearing the contig number have two CRISPR-Cas systems

A total of 13 CRISPR-Cas systems among 9 Brazilian *C. striatum* genomes and their features were listed in Table 1. Only multidrug-susceptible (MDS) isolate 1954 did not contain CRISPR-Cas system. All systems scored highly accurate (level of evidence = 4; as high as possible) based on parameters from the CRISPRFinder database, which assigns levels of evidence from 1 to 4 for spacer repetition and similarity [24]. The CRISPRFinder server identified type I-E system in all isolates. Four isolates presented two CRISPR-Cas systems, and the CRISPR-Cas system arrangement in three *C. striatum* isolates showed differences (Fig. 1a and b). All three MDR *C. striatum* isolates susceptible only to vancomycin, tetracycline, and linezolid present a novel type I-E system configuration, exhibiting a divergent gene arrangement within the *cas* operon, termed here as type I-E’ (Fig. 1b).

*C. striatum* draft or complete genomes from other countries available in the GenBank/NCBI database, including MDR 215 and 216 complete genomes [3], were used to compare with our isolates. Three draft genomes were excluded from the analysis, for presenting evidence level zero or 1 in the CRISPRFinder database. Furthermore, LK37 and 1329-caur draft genomes were not used because the CRISPR-Cas system was divided into distinct contigs. Thus, it was possible to observe that 6 *C. striatum* genomes from the GenBank/NCBI database also presented the type I-E’ system (Supplementary Table 1).

**Fig. 2:**
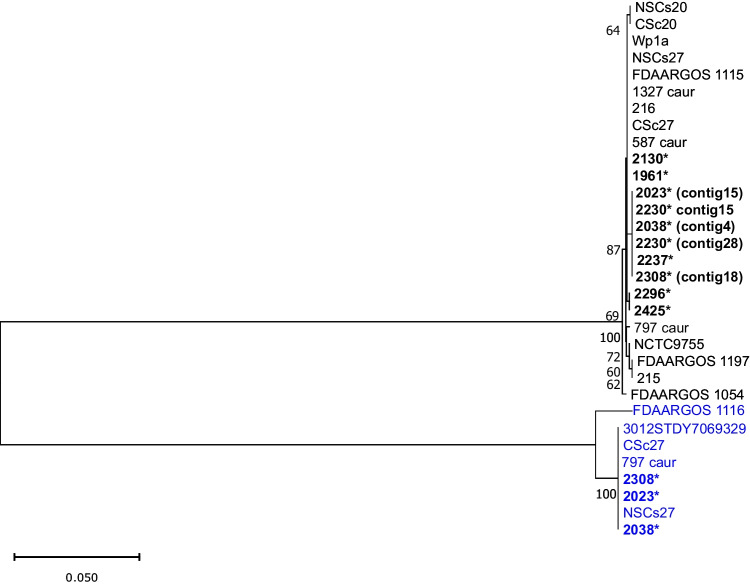
NJ tree for *cas1* gene in *C. striatum*. *Cas1* gene alignment was performed using the ClustalW algorithm in BioEdit Sequence Alignment, and the tree was generated in MegaX software. The inferred distance was calculated using p-distance. Bootstrap values (> 60%) based on 500 replicates are shown. 0.05% scale bar estimated sequence divergence. Isolates LK37 and 1329-caur were excluded from this analysis because the CRISPR-Cas system is divided into different contigs. Brazilian isolates contain an asterisk in the name. Type I-E’ and type I-E CRISPR-Cas systems were colored blue and black, respectively. FDAARGOS 1054^ T^ corresponds to *C. striatum* type strain

To further distinguish the CRISPR-Cas system in *C. striatum*, the *cas1* gene’s NJ tree was constructed. Results showed that all *cas1* genes of the type I-E CRISPR system formed a separated branch from those of the type I-E’ CRISPR system (Fig. 2). Moreover, the amino acid identities of all *cas* proteins of Brazilian *C. striatum* genomes for types I-E (*n* = 10) and I-E’ (*n* = 3) CRISPR systems were detected. Within a single type I-E or I-E’, most Cas1 proteins share more than 99% amino acid identity and some up to 100% amino acid identity. When amino acid sequences of type I-E Cas1 proteins were compared with those of type I-E’, they shared 30% of amino acid identity (Table 3).

**Table 3:** Cas protein conservation of Brazilian *Corynebacterium striatum* isolates

CRISPR type	Isolates	% amino acid identity							
		Cas3	Cse1	Cse2	Cas7	Cas5	Cas6	Cas1	Cas2
Type I-E	1961								
	2023								
	2038								
	2130								
	2230								
	2230								
	2237								
	2296								
	2308								
	2425								
	Consensus	99.7	98.6	98.7	99.8	99.6	99.6	99.9	99.8
Type I-E’	2023								
	2038								
	2308								
	Consensus	99.9	100	100	100	100	100	100	100
% amino acid identity		23.5	19.8	17.9	24.9	19.1	22.5	30.7	28.3

Geneious global alignment (Needleman-Wunsch) was used for multiple sequence alignment. For each protein, global alignment was performed within each type (I-E or I-E’), and consensus similarity between them was given in percentage

The amino acid identity percentage indicates the alignment between type I-E protein consensus sequence and type I-E’ protein consensus sequence

As *cas3* was cleaved in isolate 1961, it was not considered to calculate amino acid identity

As *cse1* was very short in isolate 2230 (contig 15) and showed a frameshift in isolate 2296, it was not considered to calculate amino acid identity

As *cas7* showed a frameshift in isolate 2038 (type I-E’), it was not considered to calculate amino acid identity

As *cas2* was broken in isolated 2023 (type I-E’), it was not considered to calculate amino acid identity

### Source of spacer sequences

A total of 640 spacer sequences were found in *C. striatum* CRISPR arrays from Brazilian isolates (Supplementary Table 2). The ViroBlast server found no hits for spacers of isolates no. 1961 and 2023. The CRISPRTarget database returned the highest number of hits (*n* = 226) among the databases used. All hits (*n* = 16) found in CRISPRCasdb returned only for CRISPR spacers found in *C. striatum* genomes with the parameters previously described in methods. About 379 spacers (59.2%) are unknown according to the database used: 73 and 306 spacers in type I-E’ and I-E systems, respectively.

The CRISPR loci found in *C. striatum* vary in length and spacer content. The longest CRISPR locus contains 117 spacers and was found in the type I-E system of MDR isolate 2308. The smallest CRISPR locus was found in MDR isolate 2023, with 9 spacers. Of the 117 spacers found in isolate 2308, five were duplicated. None of these spacer sequences was found in the additional type I-E’ system of this isolate. Forty-nine of the 117 spacer sequences were found in isolates 2230 and 2237, some with a 1 bp difference in length. A significant amount of 117 spacer sequences were found only in the type I-E system of MDR isolates 2023 and 2038, totalizing 20 and 45 spacer sequences, respectively.

The 9 Brazilian *C. striatum* isolates shared spacers with 100% similarity (Supplementary Table 3), but between type I-E’ and type I-E systems, there was no sharing of spacer sequences. Isolate 2130 was the only one that did not share spacer sequence with other isolates. The type I-E CRISPR system spacer sequences of isolate 2308 were the most shared among the isolates, with 44 of them found in the type I-E CRISPR system of isolates 2038, 2230, and 2237.

**Fig. 3:**
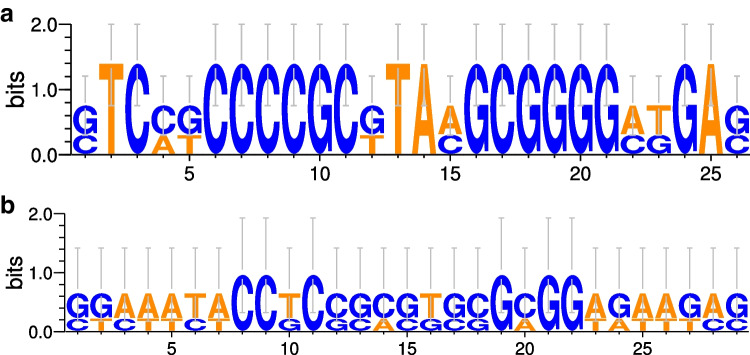
Direct repeat conservation in **a** type I-E and **b** type I-E’ CRISPR configurations of *C. striatum*. The sequence logo was created by WebLogo 2.8.2. The height of the letters shows the relative frequency of the corresponding nucleotide at that position

Repeat consensus sequences were also different among the two CRISPR system configurations (Fig. 3a and b), while they were conserved within the same layout (Supplementary Fig. 1). Within a single type I-E or I-E’, most repeat consensus shared more than 96.5% nucleotide similarity and some up to 100% nucleotide similarity. When repeat consensus nucleotide sequences of the type I-E system were compared with those of type I-E’, they shared 50% nucleotide identity.

### Genetic relationship between *C. striatum* isolates

**Fig. 4:**
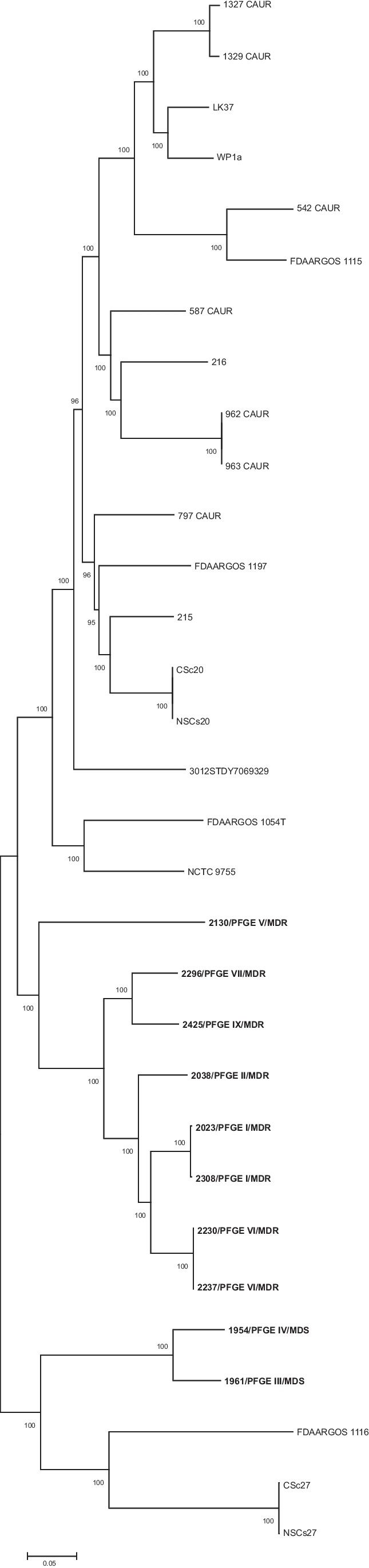
NJ tree from variation in the core genome (1619 CDS) of 31 *C. striatum* isolates. The scale bar represents the number of replacements per site. Brazilian isolates are highlighted in bold. The PFGE profiles and the strength profile according to Magiorakos et al. [23] were added. FDAARGOS 1054^ T^ corresponds to the strain of type *C. striatum*

The *C. striatum* core genome consisted of 1619 coding sequences (CDS), when calculated using 31 genomes. Phylogenetic analysis (Fig. 4) of the conserved core genome was contradictory to the type of CRISPR-Cas systems and phylogeny of *cas1* genes (Fig. 2). Furthermore, by phylogenetic analysis of the core genome, Brazilian MDR isolates were grouped in a distinct clade from MDS isolates 1954 and 1961 and from isolates of other countries (Fig. 4; Supplementary Table 1).

### Prophage regions

Isolates were submitted to PHASTER analysis for identification and annotation of prophage sequences. The results revealed the presence of 3 intact phages in isolates 1961, 2130, and 2425. An intact prophage region of 9.1 kb was identified in MDS isolate 1961, with a score of 100% containing 12 proteins. The phage with the highest number of proteins like those identified in this region was Rhodoc_Sleepyhead (GenBank NC048782_2), with 52.22% of GC content. Isolate 2130 also has a 43.2 kb intact prophage region containing 60 proteins, scoring 140%. The phage with the highest number of proteins like those in the region is Rhodoc_Jace (GenBank NC047974_15), with 54.7% of GC content. Finally, isolate 2425 showed an intact prophage region of 43.8 kb containing 64 proteins, with a score of 110%, and the most common phage found was Coryne_Poushou (GenBank NC042139_11), with 57.2% of GC content. When submitted to NCBI BLAST using nucleotide collection, the intact prophage sequence of isolate 1961 aligned with a small chromosomal region of *C. striatum* 215, about 7 kb, approximately. Of the 43.2 kb of the intact region of the prophage found in isolate 2130, about 21.7 kb were aligned with the chromosomal region of *C. striatum* strain, with correspondence with proteins commonly found on phages, such as phage tail and phage portal protein. Finally, the intact prophage region of isolate 2425 aligned only 5.4 kb, approximately, with the chromosome region of *C. striatum* DSM45711 (FDAARGOS 1197). The similarity between the spacers and the phages found in isolates 1961, 2130, and 2425 was verified. There was no match between the CRISPR spacers from isolate 1961 and the phage found in the same isolate. Four unknown CRISPR spacers from isolate 2130 matched with the phage found in this isolate. Three CRISPR spacers from isolate 2425 matched with the phage found in this isolate. Spacers 6, 10, and 31 were found in *C. simulans* (100% identity), *Gordonia* phage Easley (84.37% identity), and *Corynebacterium glyciniphilum* (87.50% identity)/Turkey Adenovirus 1 (81.25% identity), respectively.

## Discussion

*C. striatum* isolates expressing different MDR profiles have been identified as the etiologic agent of healthcare-associated infections in several countries [1, 3, 4, 7, 32] demonstrating the involvement of genetic mobile elements in the resistome of this bacterial species. *C. striatum* isolate genomes present antimicrobial resistance genes acquired, disseminated, and conserved by vertical transfer through plasmids and transposons [32]. Bacterial CRISPR-Cas systems are important elements for inhibiting horizontal gene transfer between bacterial cells [8, 33]. However, information on CRISPR-Cas systems in *C. striatum* remains unknown. In this study, we analyzed CRISPR-Cas systems, and all elements for this system (*cas* genes, repeats, leader sequences, and adjacent protospacer motifs) were characterized. In some studies, there was a reverse correlation between the CRISPR-Cas system presence and antibiotic resistance in some species such as *Enterococci* [33], but, in MDR *C. striatum*, such as *E. coli* [34], there was no significant relation.

In this study, we have identified a different configuration of the type I-E CRISPR-Cas system in 3 Brazilian isolates. To our knowledge, this is the first comprehensive study of the CRISPR-Cas system in *C. striatum*, an important MDR pathogen associated with nosocomial outbreaks in several countries [1, 3, 4, 7].

Previous studies reported the emergence of *C. striatum* isolates as novel clones by PFGE genotyping as etiologic agents of invasive infections [4, 7]. The CRISPR-Cas system was found in 9 Brazilian genomes. Of these, 8 isolates are MDR with different levels of resistance, but resistant to at least one agent in three or more antimicrobial categories [23]. Isolates 2023, 2038, and 2308, classified as PFGE profiles I and II, are the most resistant, susceptible only to tetracycline, vancomycin, and linezolid [4] (Table 2). It is important to emphasize that these 3 more resistant isolates presented an additional system with an alternative arrangement named here type I-E’. Although the resistance profiles of *C. striatum* isolates from other countries used in this study are unknown, some showed the type I-E’ CRISPR-Cas system too (Supplementary Table 1). A similar configuration was found in *C. diphtheriae*, the causative agent of diphtheria disease, named type I-Ea [8, 35]. In this case, the CRISPR loci are located between the *cas3* and *cse1* genes.

This study revealed a diversity of spacers among CRISPR arrays of Brazilian *C. striatum* genomes. Of the 640 spacers, 113 were shared among the 9 isolates, being 104 spacers between the type I-E system and 9 spacers between the type I-E’ system. There was no sharing of spacers between the two types of systems. CRISPR loci found in our isolates presented a significant amount of spacer sequences (*n* = 135), with similarities ranging from 81.2 to 100% to prophage of *Rhodococcus* (phage Rhodoc REQ3—GenBank NC016654), belonging to family Siphoviridae, isolated from wastewater. This prophage is also found in the *C. striatum* type strain. Some spacers showed similarities above 81.2% with corynebacteriophages or unannotated regions of other *Corynebacterium* species, such as *Corynebacterium aurimucosum*, *Corynebacterium simulans*, and *Corynebacterium ulcerans*. Eleven spacer sequences from our Brazilian isolates, except for isolates 1961, 2296, and 2425, matched with *Corynebacterium* phage phi673 (GenBank NC042354), a lytic phage of *Corynebacterium glutamicum* ATCC 13,032.

Thirteen spacer sequences matched with *C. striatum* 215, 216, and type strain by the CRISPR-Cas +  + database, with similarities ranging from 84.3 to 100%. Interestingly, 5 spacers showed similarity with non-corynebacteria plasmids, such as *Bacillus*, *Sinorhizobium*, and *Rhodobacter*, and the last had the same score as the phage Salmon SPN1S spacer. This diversity of spacers may reflect a history of previous invasions, with hits ranging from uncultivable viruses, *Rhodococcus*, *Mycobacterium*, *Gordonia*, and *Corynebacterium* phages to plant pathogenic bacteria plasmids, such as *Ralstonia solanacearum*, although for the majority (59.7%) no hits were found in the databases, indicating that there is a reservoir of unexplored plasmids and corynebacteriophages.

Isolates 2296 and 2425 shared 3 spacers with 84% similarity to *C. simulans* chromosome region. Moreover, spacer 26 from isolate 2296 had similarity to phage *Propionibacterium* PFR1 found in isolate 2425 (spacer 22) (Supplementary Table 3). Some type I-E’ system spacers from isolate 2038 (*n* = 5) and type I-E system spacers from isolates 2130 (*n* = 1), 2296 (*n* = 2), and 2308 (*n* = 5) were duplicated within the CRISPR arrays. This event has also been reported in *Corynebacterium diphtheriae*, in which 9 duplicated spacers were found within CRISPR arrays [8]. The relationship between spacer duplication and increased efficiency of CRISPR-Cas-mediated immunity against invasive DNA is controversial [8].

It is important to emphasize that the total of 640 spacers are those that were present near the CRISPR-Cas system. Except for isolates 2038 and 2296, there are other CRISPR arrays located in other regions of the genomes. Therefore, there may be more than 113 spacers shared between the 9 Brazilian isolates. The presence of mobile genetic elements, such as insertion sequences close to some CRISPR-Cas loci, may indicate that these systems may have been acquired horizontally. This observation is supported by the lower DNA GC content of *cas* operons (53% for both type I-E and type I-E’ systems) when compared with the average GC content of the *C. striatum* genome (59.3%). Furthermore, in 6 isolates, the *cas* operons in both types I-E and I-E’ systems are close to transposases of families 21, 30, and 481 (Fig. 1a and b). The IS21 element is carried by plasmid IncP R68 and is close to the kanamycin resistance gene therein [36]. IS30 works on a structure of compound transposons and has been identified as part of compound transposons that flank the colistin resistance gene mcr-1 [37]. The IS481 family transposase actively mobilizes the TnRErm46 transposable element that contains the *erm* gene responsible for emerging macrolide resistance [38]. According to Sangal et al. [8], the type I-E system is commonly flanked by mobile genetic elements, indicating the potential mobility of this system between isolates.

According to Ramos et al. [4], Brazilian isolates 2230 and 2237 are clones belonging to PFGE profile VI and were isolated from the same patient with an interval of 1 month in 2011 (Table 1). However, both isolates showed differences in the number of CRISPR-Cas systems. Isolate 2230 presented two type I-E CRISPR-Cas systems, and isolate 2237, only one type I-E CRISPR-Cas system. One of the CRISPR-Cas systems of isolate 2230 has 47 spacer sequences, as well as the number of spacers found in the CRISPR-Cas system of isolate 2237. The 47 spacer sequences found in both clones of the same PFGE profile are similar to each other (Supplementary Table 3).

The leader sequence is adjacent to the first spacer of the CRISPR locus and acts as a promoter for locus transcription and as a guide for novel spacer incorporation [39, 40]. This is a region rich in AT (adenine and thymine) and in Brazilian isolates that ranged from 44 to 58%, and no difference between %AT of the leader sequence of the type I-E system and the type I-E’ system was observed. The leader sequence of isolates 2296, 2308 (contig 18) and isolate 2425 shared 100% similarity (Fig. 1a) as well as the leader sequence of two CRISPR-Cas systems of isolate 2237.

The core genome is defined as the content of genes present in all representatives of a species [41]. Core genome analysis was performed to verify if there was a correlation between the CRISPR system type and the core genome. Our core genome results showed that there is no such correlation. Additionally, there was no correlation between the core genome and the *cas1* gene phylogeny (Fig. 2) and direct repeat consensus (Supplementary Fig. 1). This fact has already been observed for the *C. diphtheriae* species [8]. By the core genome phylogenetic analysis, MDS 1954 and 1961 isolates were grouped with one pre-1992 surgical incision isolate and two Chinese sputum and nasopharyngeal isolates in 2018. MDR isolates 2023 and 2308, classified as PFGE profile I in previous studies [4, 7], were grouped in the same clade. Similarly, MDR isolates 2230 and 2237, classified as PFGE profile VI [4], were grouped in the same clade. *C. striatum* Brazilian MDR isolates are grouped into a distinct clade from those isolates from other countries, but we do not know their antimicrobial susceptibility profiles, except for MDR isolates 215 and 216 from the USA mentioned above [3].

Searching for the CRISPR system of the Cas1 protein type I-E’ by BLASTp/NCBI, we have found that it exists only in one other *Corynebacterium* species: *C. simulans* (data not shown), phylogenetically related to *C. striatum*. Also, *cas* operon arrangement in the *C. simulans* genome is equivalent to the type I-E’ system found in our *C. striatum* isolates. When all type I-E system Cas proteins were compared with those of type I-E’ system, they showed low identity (Table 3). In addition to the low similarity (30.7%) between the type I-E and type I-E’ system Cas1 proteins (Table 3), phylogenetic analysis revealed that the type I-E’ system Cas1 protein branched independently from the type I-E system Cas1 protein (Fig. 2).

Cas3 protein is the signature of type I systems, responsible for target DNA cleavage and degradation [43]. *Cas3* gene (Supplementary Fig. 2) phylogenetic analyses were also carried out and showed that the *cas3* gene of the isolates that have the type I-E’ system separated into a different clade from the isolates that have the type I-E system.

Horizontal gene transfer favors the survival and adaptation of bacteria and archaea that acquire virulence factors, the ability to degrade toxic compounds, and antibiotic resistance. Although CRISPR systems provide bacterial immunity against horizontally acquired elements, this system is not 100% effective. Some acquired elements are maintained when they confer selective advantage [40], and this could explain the presence of intact prophages found in 3 of our isolates. To determine whether a bacterial lineage has been previously exposed to a specific phage, a matching CRISPR spacer sequence must be found. Phages can regulate host population size and can alter bacterial physiology as well bacteria phenotype in toxin production, antibiotic resistance genes, virulence factors, photosystem components, CRISPR-Cas system, and other metabolic and genes with unknown functions [43]. The intact prophage regions of isolates 2130 and 2425 are similar in length, 43.2 kb and 43.8 kb, respectively, both do not share sequence similarity and also differ in GC content. A large part of the prophage region is found in isolate MDS 1961 (6.9 kb to 9.1 kb), aligned with one of the 4 intact prophage regions *C. striatum* 215, an MDR sputum isolate from the USA. Approximately 21 kb of the phage region of isolate 2130 was aligned with various phage regions found in *C. striatum* strain, and only approximately 5 kb of the phage region was found in isolate 2425, aligned with the phage region of *C. striatum* DSM 45,711 (FDAARGOS 1197), isolated from blood in 2011 in Italy, according to information available in the German Collection of Microorganisms DSMZ.^10^ In addition to the intact phages found in 3 of our isolates previously described, all isolates showed incomplete phage sequences with a total of 8 in isolate 2308 (data not shown). These finds show that the CRISPR-Cas system is not always effective against foreign DNA invasions.

As important emergent pathogens, the CRISPR-Cas system in *C. striatum* was found in 9 genomes from a nosocomial outbreak that occurred at a public hospital in Rio de Janeiro, Brazil. Of these 9 clinical isolates, 8 are MDR with different resistance levels. According to the current classification method [11], the 13 CRISPR-Cas loci detected in our isolates are classified as type I-E system. However, additional *cas* operon alternative gene arrangement in 3 isolates (Fig. 1b) was distinct from the classic type I-E system, despite the low identity between all proteins of both systems (Table 3). Phylogenetic analyses of *cas1* (Fig. 2) and *cas3* genes (Supplementary Fig. 2) and of direct repeat consensus sequences (Supplementary Fig. 1) separated both type I-E and type I-E’ systems into two distinct clades. Furthermore, no spacers were shared between the two systems. As observed by Ramos et al. [4], the profile PFGE I was the most frequently observed in the hospital, with susceptibility only to tetracycline, vancomycin, and linezolid, and it also presented a second CRISPR-Cas system called here type I-E’ system. As observed for other species, such as *E. coli* and *Enterococci* [15, 16], there is no correlation between the presence of CRISPR-Cas system and multidrug resistance in *C. striatum*. This is the first study about the CRISPR-Cas system occurrence, arrangement, and diversity in *C. striatum*, and these finds may contribute to further investigations, in particular about the role of the CRISPR-Cas system in *C. striatum*.

## Supplementary Information

Below is the link to the electronic supplementary material.

Supplementary file1 (PDF 38 KB)

Supplementary file2 (PDF 43 KB)

Supplementary file3 (DOCX 20.2 KB)

Supplementary file4 (XLSX 25.0 KB)

Supplementary file5 (XLSX 12.0 KB)

Supplementary file6 (DOCX 13 KB)

## Data Availability

All data generated or analyzed during this study are included in this published article (and its supplementary information files).

## Footnotes

1 https://crisprcas.i2bc.paris-saclay.fr/CrisprCasFinder/Index

2 http://crispr.otago.ac.nz/CRISPRTarget/crispr_analysis.html

3 https://indra.mullins.microbiol.washington.edu/viroblast/viroblast.php

4 https://weblogo.berkeley.edu/logo.cgi

5 http://phaster.ca/

6 https://github.com/tseemann/prokka/issues

7 https://sanger-pathogens.github.io/Roary/

8 https://www.megasoftware.net/

9 https://www.geneious.com

10 https://www.dsmz.de/collection/catalogue/details/culture/DSM-45711
