# Supplementary figures and images for: Novel configurations of type I-E CRISPR-Cas system in Corynebacterium striatum clinical isolates
Source: Braz J Microbiol. 2022 Dec 7;54(1):69–80. doi: 10.1007/s42770-022-00881-4 (PMC9944170; doi:10.1007/s42770-022-00881-4)

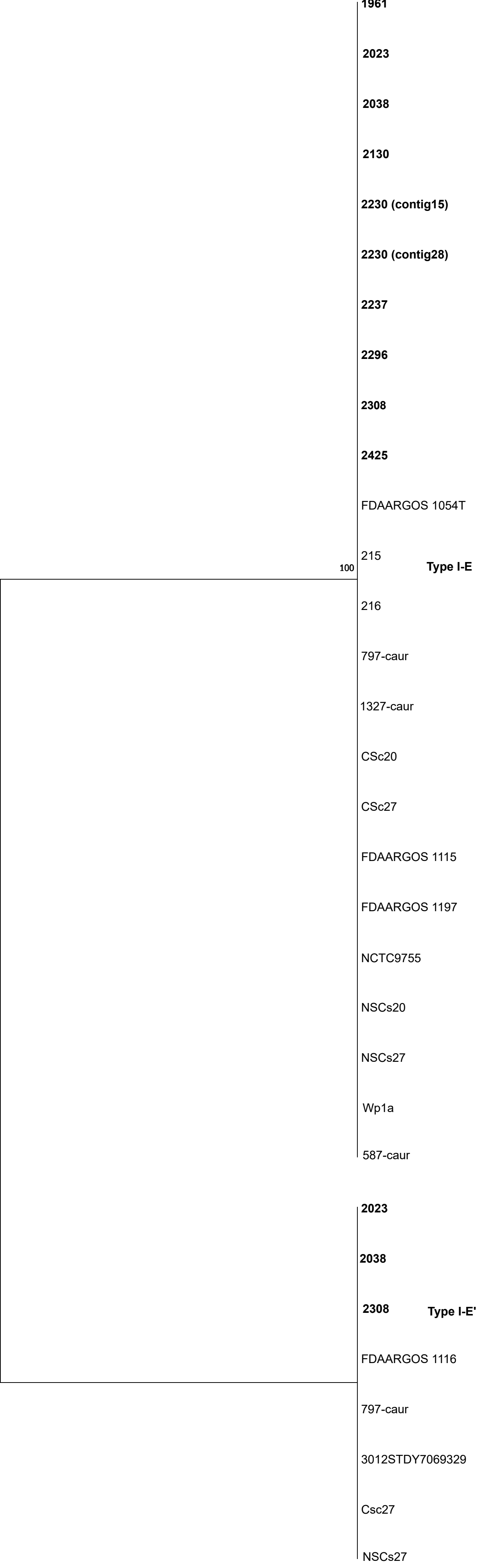

Supplement: Supplementary file 1 — Supplementary file1 (PDF 38 KB) [file 42770_2022_881_MOESM1_ESM.pdf]

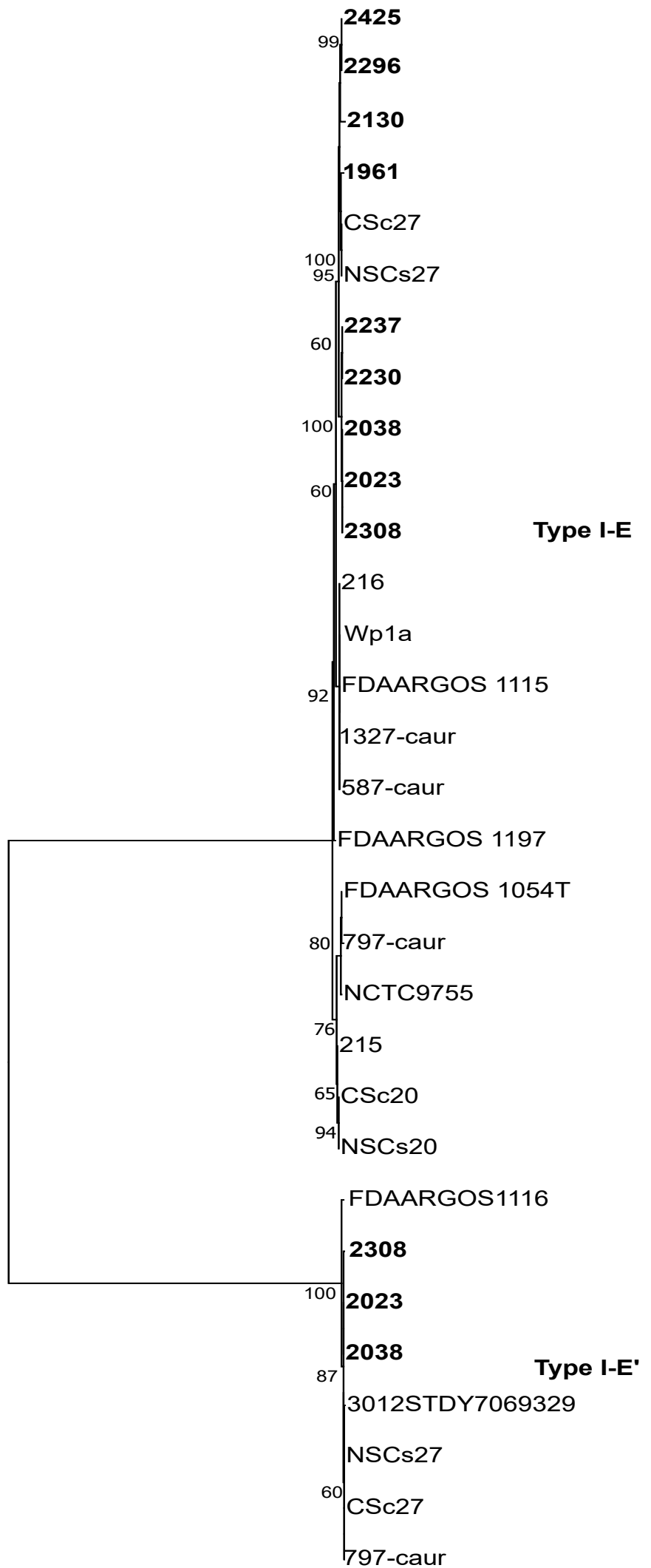

Supplement: Supplementary file 2 — Supplementary file2 (PDF 43 KB) [file 42770_2022_881_MOESM2_ESM.pdf]
