# Supplementary material for: Novel configurations of type I-E CRISPR-Cas system in Corynebacterium striatum clinical isolates
Source: Braz J Microbiol. 2022 Dec 7;54(1):69–80. doi: 10.1007/s42770-022-00881-4 (PMC9944170; doi:10.1007/s42770-022-00881-4)
Supplement: Supplementary file 3 — Supplementary file3 (DOCX 20.2 KB) [file 42770_2022_881_MOESM3_ESM.docx]

| **Supplementary Table 1.** Details of CRISPR-Cas loci in *C. striatum* isolates and type strain. | | | | | |
| --- | --- | --- | --- | --- | --- |
| **Isolates** | **Year of isolation** | **Isolation sites** | **Country** | **Crispr-Cas system**  **(nº of spacers)** | **GenBank**  **accession nº** |
| 542-caur | NI | sputum | USA | I-E’ (1)* | JVCW00000000.1 |
| 587-caur | NI | BAL | USA | I-E (18) | JVBA00000000.1 |
| 797-caur | NI | NI | USA | I-E (5)  I-E’ (45) | JUSN01000012.1 |
| 962-caur | NI | fluid | USA | I-E (2)* | JULW01000001.1 |
| 963-caur | NI | fluid | USA | I-E (2)* | JULV01000001.1 |
| 1327-caur | NI | NI | USA | I-E (99) | JVTN01000001.1 |
| 1329-caur | NI | NI | USA | I-E (20) | JVTL00000000.1 |
| 3012STDY  7069329 | 1989 | knee aspirate fluid | NI | I-E’ (80) | CAACYF000000000.1 |
| CSc20 | 2018 | sputum | China | I-E (92) | VCPB00000000.1 |
| CSc27 | 2018 | sputum | China | I-E (150)  I-E’ (34) | VCOZ00000000.1 |
| FDAARGOS  1054^T^ | Before 1990 | NI | NI | I-E (75) | NZ_CP066290.1 |
| FDAARGOS  1115 | Before 1992 | eye | NI | I-E (108) | NZ_CP068158.1 |
| FDAARGOS  1116 | Before 1992 | leg infection from surgical incision | NI | I-E’ (60) | NZ_CP068157.1 |
| FDAARGOS  1197 | 2011 | blood | Italy | I-E (224) | NZ_CP069514.1 |
| LK37 | 2011 | foot ulcer | USA | I-E (81) | RAQW00000000.1 |
| NCTC9755 | NI | NI | NI | I-E (105) | UFYO00000000.1 |
| NSCs20 | 2018 | nasopharynx | China | I-E (92) | VCPA00000000.1 |
| NSCs27 | 2018 | nasopharynx | China | I-E (27)  I-E’ (34) | VCOY00000000.1 |
| Wp1a | 2013 | blood | USA | I-E (72) | SBIF00000000.1 |
| 215 | 2016 | sputum | USA | I-E (104) | NZ_CP024931.1 |
| 216 | 2016 | tracheal aspirate | USA | I-E (75) | NZ_CP024932.1 |
| * CRISPR array with evidence level = 1;  BAL, bronchoalveolar lavage;  NI, not informed;  T, type strain of *C. striatum* DSMZ 20668;  USA, United States of America;  FDAARGOS1054^T^ corresponds to *C. striatum* DSM 20668 type strain. | | | | | |

**Novel configurations of type I-E CRISPR-Cas system in *Corynebacterium striatum* clinical isolates.**

Brazilian Journal of Microbiology.

Authors: Juliana Nunes Ramos*, Paulo Victor Pereira Baio; João Flávio Carneiro Veras; Érica Miranda Damásio Vieira; Ana Luiza Mattos-Guaraldi; Verônica Viana Vieira

* Correspondence to Dr. Juliana Nunes Ramos/LDCIC/UERJ/E-mail: jnr.uerj@gmail.com
