# Supplementary material for: Novel configurations of type I-E CRISPR-Cas system in Corynebacterium striatum clinical isolates
Source: Braz J Microbiol. 2022 Dec 7;54(1):69–80. doi: 10.1007/s42770-022-00881-4 (PMC9944170; doi:10.1007/s42770-022-00881-4)
Supplement: Supplementary file 6 — Supplementary file6 (DOCX 13 KB) [file 42770_2022_881_MOESM6_ESM.docx]

**Supplementary Figure 1**. Phylogenetic tree for repeat direct consensus nucleotides (32 positions) in *C. striatum*. The DR consensus nucleotides alignment was performed using the ClustalW algorithm in BioEdit Sequence Alignment and Neighbor-Joining tree were generated in MegaX. The inferred distance was calculated using the p-distance method. Bootstrap values (> 60 %) based on 500 replicates are shown. Bar 0,05 % estimated sequence divergence. The LK37 and 1329-caur isolates were excluded from this analysis because the CRISPR-Cas system was divided into different contigs. The CRISPR arrays of the 542-caur, 962-caur, and 963-caur isolates had an evidence level = 1 in the CRISPRFinder database, therefore they were not considered in this analysis. Brazilian isolates are highlighted in bold. FDAARGOS 1054^T^ corresponds to *C. striatum* type strain (=DSM 20668).

**Supplementary Figure 2**. Phylogenetic *cas3* gene in *C. striatum*. The *cas3* genes alignment was performed using the ClustalW algorithm in BioEdit Sequence Alignment and Neighbor-Joining tree was generated in MegaX. The inferred distance was calculated using the p-distance method. Bootstrap values (> 60 %) based on 500 replicates are shown. Bar 0,05 % estimated sequence divergence. The LK37 and 1329-caur isolates were excluded from this analysis because the CRISPR-Cas system was divided into different contigs. The CRISPR-Cas systems of the 542-caur, 962-caur, and 963-caur isolates had an evidence level = 1 in the CRISPRFinder database, therefore they were not considered in this analysis. Brazilian isolates are highlighted in bold. FDAARGOS 1054^T^ corresponds to *C. striatum* type strain (=DSM 20668).

**Supplementary Table 2.** Hits found to spacer sequences in the CRISPRTarget, ViroBlast, and CRISPR-Cas databases. For each hit, the number refers to the spacer number, the similarity found in percentage, and the score. Zero = no hits found for the parameters used described in the methodology.
